# Application of clitoris exposure + episioplasty + dermabrasion + platelet-rich plasma injection + chemexfoliation in vulvar lichen sclerosus

**DOI:** 10.3389/fped.2023.1276786

**Published:** 2023-12-04

**Authors:** Xianhui Shang, Changmei Chen, Hong Ma, Peng Zhao, Yuchen Mao, Hong Liu, Cao Wang, Kaiyi Mao, Zhen Luo, Yingbo Li, Guangxu Zhou, Hongyang Tan

**Affiliations:** ^1^ Department of Pediatric Surgery, Guizhou Children’s Hospital, Zunyi, China; ^2^ Department of Pediatric Surgery, Affiliated Hospital of Zunyi Medical University, Zunyi, China; ^3^ Department of Physiology, Basic Medical College, Medicine & Technology College of Zunyi Medical University, Zunyi, China

**Keywords:** clitoris exposure, episioplasty, dermabrasion, PRP injection, chemexfoliation, VLS, therapeutic effect

## Abstract

**Introduction:**

To investigate the therapeutic effect of clitoris exposure + episioplasty + dermabrasion + platelet-rich plasma (PRP) injection + chemexfoliation on vulvar lichen sclerosus (VLS).

**Methods:**

Twenty children with VLS (under 14 years old) at our hospital from July 2020 to November 2022 were enrolled and treated with clitoris exposure + episioplasty + dermabrasion + PRP injection + chemexfoliation. Additionally, symptomatic changes and improvements in signs were recorded.

**Results:**

Significant therapeutic effects were achieved in all children enrolled in this study. The Cattanco score was 8.02 ± 1.22 points before surgery, 2.21 ± 0.70 points 3 months after surgery, and 2.61 ± 0.59 points 6 months after surgery, demonstrating that the score after surgery was significantly lower than that before surgery (*p* < 0.05). Mild complications (one case of mild vulvar swelling, one case of minor bleeding, and one case of superficial ulcer) were observed in three children after surgery, with an overall complication incidence of 15%; all complications were improved after the intervention, and no severe adverse reactions were observed. Recurrence was observed in one child (5%) 6 months after surgery.

**Conclusion:**

Clitoris exposure + episioplasty + dermabrasion + PRP injection + chemexfoliation is an effective approach for the treatment of VLS.

**Systematic Review Registration:**

https://www.chictr.org.cn/searchproj.html, identifier: ChiCTR2100054787.

## 1. Introduction

Vulvar lichen sclerosus (VLS), a common disease of the external genitalia, is characterized by inflammation, itching, atrophy, and sclerosis of the vulvar skin (1). The disease often progresses chronically with recurrent episodes. Without timely and appropriate treatment, severe complications can develop, including further atrophy, adhesion, and scar formation in the vulvar region. These changes can lead to a loss of normal anatomy and function, as well as an increased risk of local cancer. While VLS is relatively easy to diagnose, its treatment poses significant challenges. Early diagnosis and intervention are crucial for improving long-term patient outcomes. It mainly occurs in females and severely affects their quality of life and psychological health; when it occurs in children and adolescents, it is very harmful to their physical and mental development.

At present, general treatment, drug therapy, physical therapy, and operative therapy are the main treatment strategies for VLS (2). General treatment often involves the application of topical moisturizing lubricants such as cod liver oil ointment and vitamin E cream. These are used as long-term maintenance therapies to improve the local skin barrier function and alleviate symptoms like vulvar dryness.

For drug therapy, topical glucocorticoids are the first-line treatment for VLS. The treatment with glucocorticoids is divided into two stages: induction of remission and maintenance therapy. Initially, during the induction phase, a topical glucocorticoid ointment or cream is applied for a period of 3–4 months. Following this phase, the maintenance stage involves the continued use of a local low-dose glucocorticoid ointment or cream. This lifelong maintenance helps control symptoms, reduce the recurrence rate, and lower the risk of vulvar adhesion formation and malignant transformation.

Physical therapy options, including vulvofocused ultrasound, dot-matrix laser, and photodynamic therapy, are gaining popularity as alternative treatments due to their safety, effectiveness, and minimally invasive nature.

In cases where conservative treatments are unsuccessful, and particularly in instances of vulvar adhesion, surgical treatment may be considered. This can involve procedures ranging from local focus excision to simple vulvectomy, depending on the severity and specifics of the case.

However, traditional treatment approaches have several limitations. For instance, the efficacy of drug therapy is inconsistent, and the disease is prone to recurrence; additionally, physical therapy and operative therapy can cause trauma and pain (3). Therefore, more effective and convenient clinical treatment approaches are urgently needed. A therapeutic regimen combining clitoris exposure, episioplasty, dermabrasion, platelet-rich plasma (PRP) injection, and chemexfoliation has received increasing attention in recent years (4, 5) and has shown strong therapeutic effects, effectively improving the signs and symptoms of VLS and resulting in a low recurrence rate. We found that relevant technologies have been applied in clinical practice, (1) the clinical application of autologous PRP. For example, autologous PRP promotes healing at the skin donor site; (2) the clinical application of skin grinding. For example, the dose-effect relationship of afentanil combined with propofol for painless skin grinding in children. (3) Because Vulvar Lichen Sclerosus can cause incomplete exposure of the clitoris and adhesion of the vulva, we perform clitoris exposure and episioplasty to improve the shape of the vulva and remove the diseased tissue. This is a routine gynecological surgery for children. (4) Chemical exfoliation has been widely used in the treatment of various dermatological diseases and skin rejuvenation, and a large number of studies at home and abroad have confirmed its clinical safety and effectiveness (6–10). However, few studies have assessed its application in the treatment of VLS. Therefore, this study explored the clinical efficacy and safety of this therapeutic regimen for VLS to further expand the therapeutic options for the disease.

## 2. Participants and methods

### 2.1. Study participants

**Figure 1:**
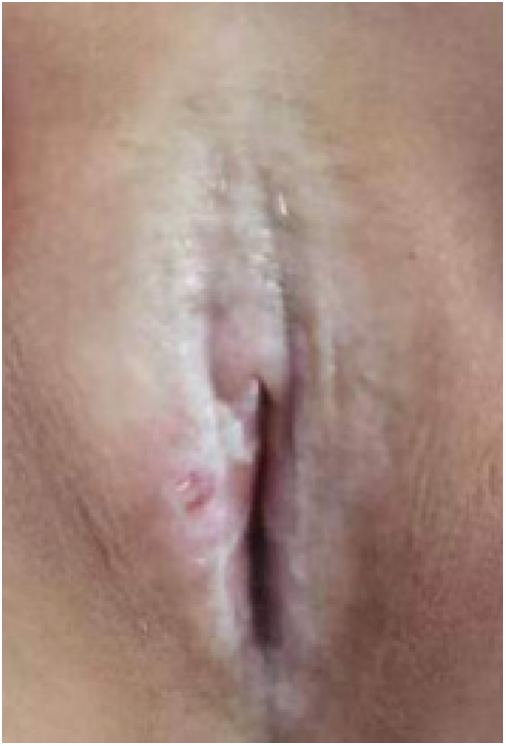
VLS case 1.

**Figure 2:**
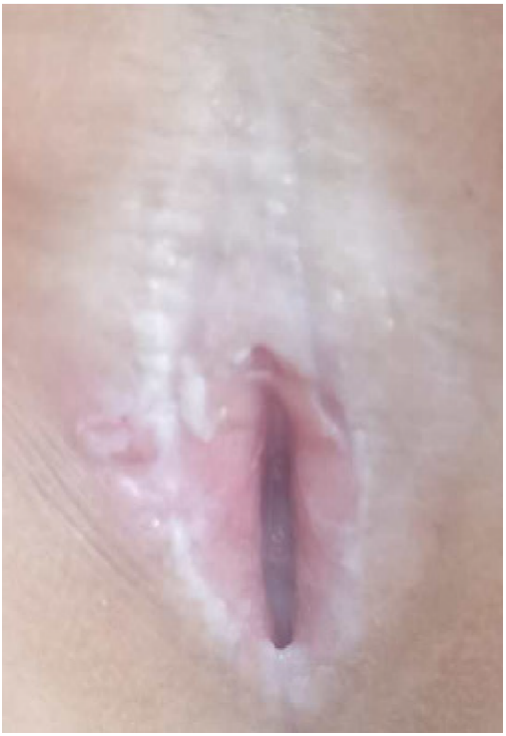
VLS case 2.

**Figure 3:**
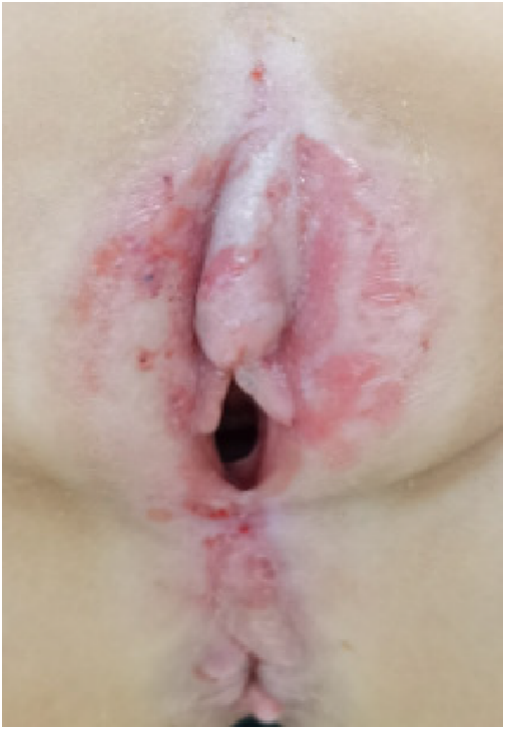
VLS case 3.

Twenty children with VLS who received treatment in our hospital from July 2020 to November 2022 were enrolled (e.g., Figures 1–3). The inclusion criteria were as follows: (1) a diagnosis of VLS according to the diagnostic criteria (11) (i.e., symptoms of inflammation, itching, atrophy, and sclerosis of the vulvar skin, no urethral and vulvar lesions, and a definitive diagnosis of VLS through histopathology and immunohistochemistry); (2) age of 5–14 years; (3) no previous operative therapies; (4) Serious impact on children's life and sleep, and failure of conservative treatment; and (5) voluntarily participation in the study and completion of the informed consent form by the patient and their family. Children were excluded from enrolment if they had vulvitis and vulvar sclerosis caused by other factors including mental illness, resulting in an inability to cooperate with the treatment and care, severe complications (such as severe infections and bleeding tendencies), or major organic diseases (malignant tumors, cardiovascular, cerebrovascular diseases, etc.). This study was approved by the local ethics committee, ethics number: KLL-2023-393.

### 2.2. Methods

**Figure 4:**
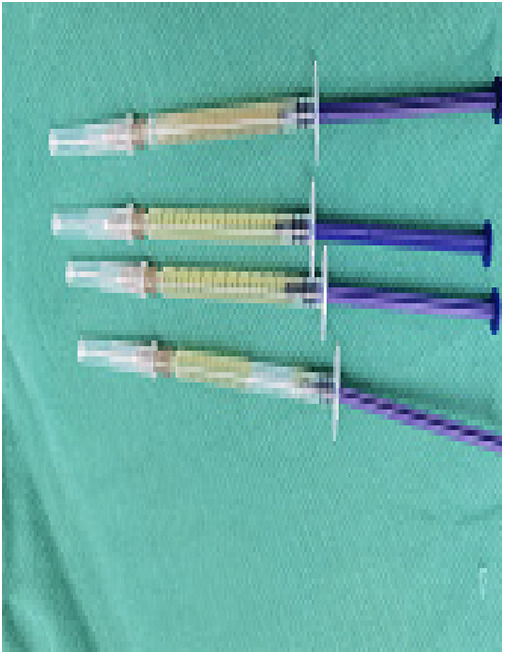
PRP.

**Figure 5:**
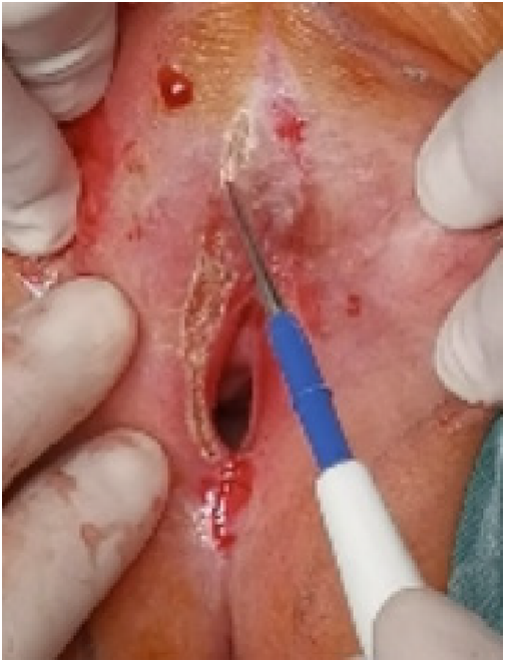
Clitoris exposure.

**Figure 6:**
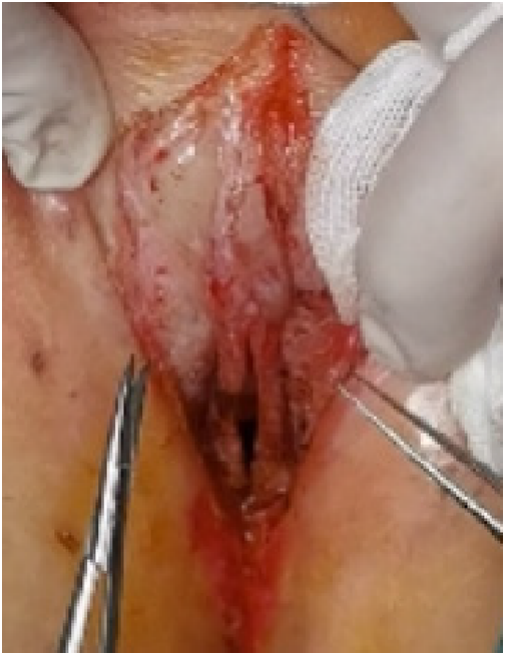
Episioplasty.

**Figure 7:**
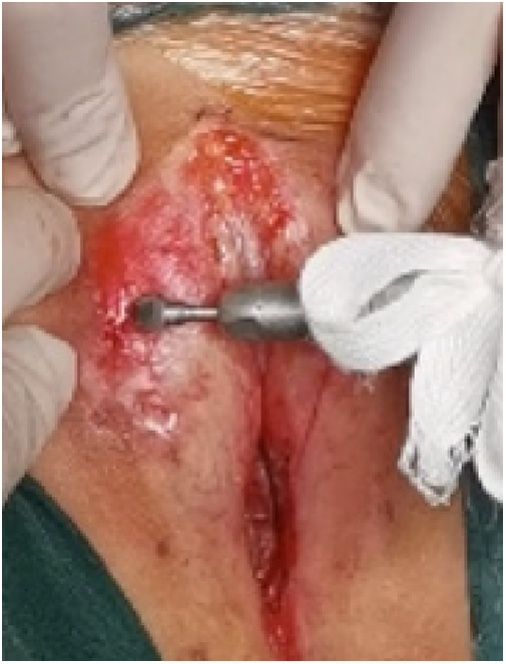
Dermabrasion.

**Figure 8:**
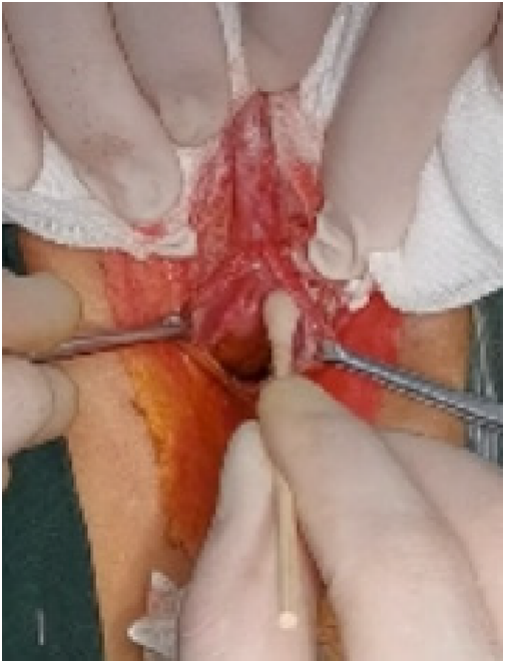
Exfoliation.

**Figure 9:**
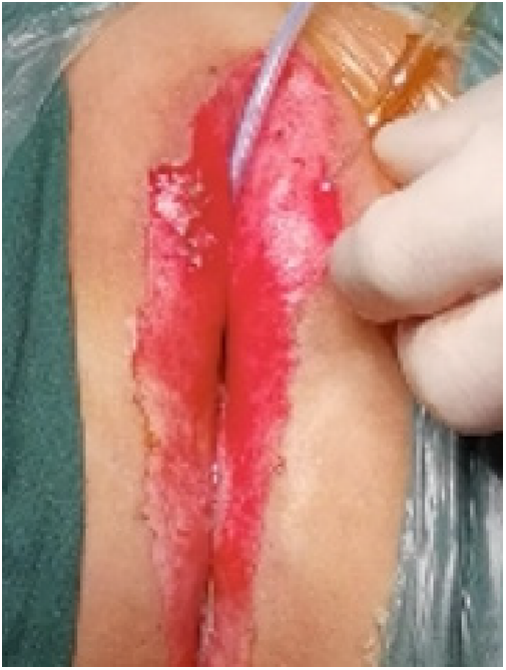
PRP was injected into the damaged vulva area.

**Figure 10:**
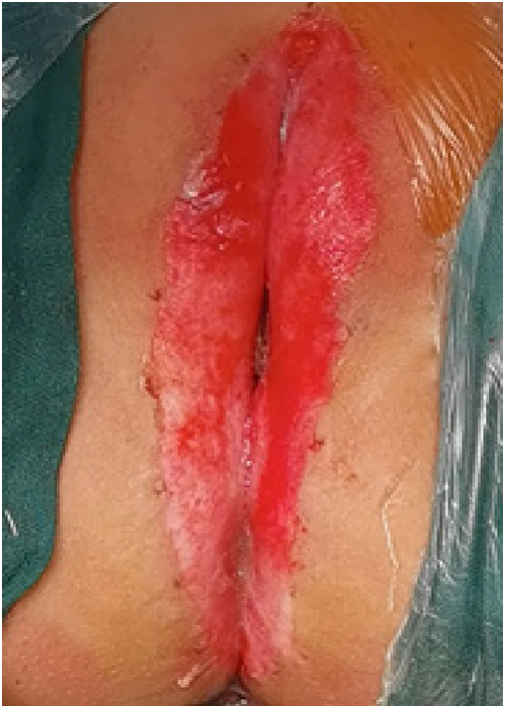
Appearance 1 day after surgery.

**Figure 11:**
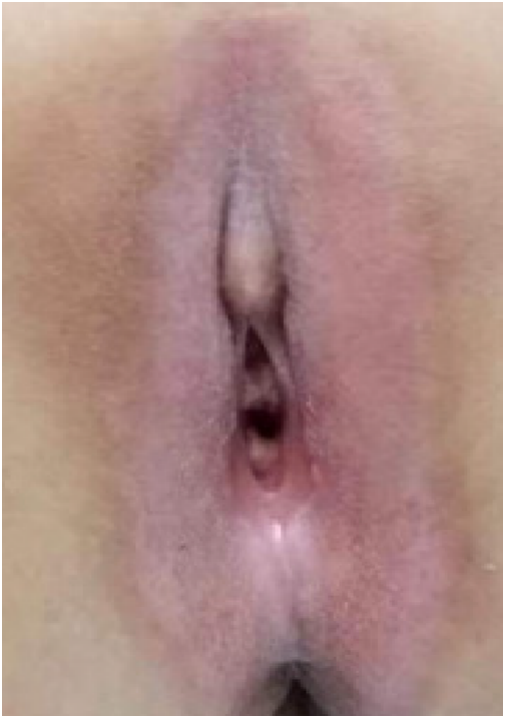
Appearance 3 months after surgery.

**Figure 12:**
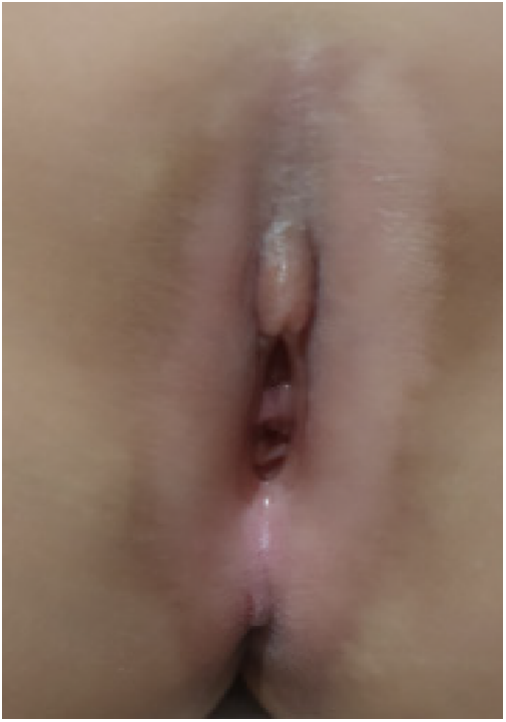
Appearance 6 months after surgery.

(1) Preparation of PRP: Peripheral blood (15–30 ml, depending on the age and weight of the child) was collected and centrifuged in a centrifuge with a horizontal rotor at 350–600 × g for 10 min. After the blood sample was divided into three layers (yellow layer, buffy coat, and red layer from top to bottom), the whole yellow layer and an appropriate amount of the buffy coat were transferred to a new centrifuge tube and centrifuged again at 1,500 × g for 20 min. Thereafter, the upper layer of the blood sample [i.e., the platelet-poor plasma (PPP) layer] was extracted for later use, and the lower layer (i.e*.*, the PRP) was mixed by oscillation with a platelet enrichment factor of 5.1 ± 1.3 times (Figure 4). (2) Procedures of clitoris exposure + episioplasty + dermabrasion + PRP injection + chemexfoliation: (1) Clitoris exposure: Following successful general anesthesia, children were placed in the supine position, followed by disinfection and draping. The bladder was then emptied using a catheter, and the tumescent solution was injected locally. After the tissues became tumescent, the adhesion line was identified, along which a 1 cm incision was then made with an electric knife to expose the glans of the clitoris (Figure 5). (2) Episioplasty: After exposing the clitoris, episioplasty (aiming to improve the shape of the vulva and removing diseased and deformed tissues) was implemented using knives and suture materials as follows. The tumescent solution was injected into the right labium majus pudendi to reveal the adhesion line, which was cut open (to a depth of 1.5 cm) with a needle electrode to separate the labia majora and labia minora grooves. The same procedures were performed on the left side (Figure 6). (3) Dermabrasion, PRP injection, and chemexfoliation: Following the local injection of the tumescent solution, the superficial layer of lesions was locally scrapped off with a 15-gauge knife, and the corneum, dermis, and pathological layer were then removed using a dermabrasion machine (Figure 7). Thereafter, exfoliation was carried out using a preparation solution in areas inaccessible to dermabrasion (containing 88% phenol, croton oil, hexachlorophenol liquid soap, and distilled water) (Figure 8), while non diseased areas in children such as the urethra and vagina should be protected. Finally, PRP was injected into the damaged vulva area to promote wound healing and tissue regeneration (Figure 9). (4) At the end of the procedures, erythromycin ointment was topically applied, and the wound was covered with crushed gelatin sponge and petrolatum gauze. Erythromycin ointment was then applied daily to the vulva for one week post-surgery to prevent infection and re-adhesion. One week after surgery, the urinary catheter was removed, and all children were followed up. There were no urinary difficulties or urinary tract infections for children before and after surgery. The vulvar appearance the day after surgery is shown in Figure 10, with follow-up appearances at three months and six months presented in Figures 11, 12, respectively.

### 2.3. Observation indexes

(1) The age, body mass index, course of disease, and menstrual period of all children were summarized. (2) The therapeutic effect was assessed before surgery and 3 and 6 months after surgery with the Cattanco score (12), which assesses four aspects (itching degree, skin color, skin elasticity, and extent of disease). Each aspect is assigned a score of 0 to 3 points, resulting in a total score out of 12 points; a higher score indicates more severe symptoms. (1) Itching degree was classified as none (0 points), mild (1 point), moderate (2 points), and severe (3 points). (2) Skin elasticity was classified as normal (0 points), slightly poor (1 point), thin (2 points), and chapped (3 points). (3) Skin color was classified as normal (0 points), red (1 point), pink (2 points), and white (3 points). (4) The extent of disease was classified as 0% (0 points), less than 30% (1 point), 30%–50% (2 points), and more than 50% (3 points). (3) All children were observed to detect any postoperative complications, and a 6-month follow-up survey was conducted to assess the recurrence rate within this period.

### 2.4. Statistical analysis

All data were processed by SPSS 20.0 software. Enumeration and measurement data were described by [*n* (%)] and (*χ *± *s*), respectively. Perioperative measurement data were compared with paired *t*-tests, and the significance level was set at *α* = 0.05.

## 3. Results

### 3.1. General data of children

The 20 children were 5–14 (8.36 ± 2.02) years old, with a weight of 14.54–56.20 (29.00 ± 9.70) kg and a course of disease of 12–28 (18.90 ± 2.72) months. Two children (10%) had autoimmune thyroid diseases, and five children (25%) had a family history of VLS.

### 3.2. Comparison of Cattanco score

The Cattanco score comparison. The scores of Cattanco subscale and the total score at 3 and 6 months after surgery were significantly lower than those before surgery (*p *< 0.05), whereas the scores were comparable at 3 and 6 months after surgery (*p* > 0.05) (Table 1).

**Table 1:** Comparison of Cattanco score of children before and after surgery (points).

Indexes	Before surgery	3 months after surgery	6 months after surgery
Itching degree	2.51 ± 0.29	0.69 ± 0.09^a^	0.72 ± 0.05^a^
Skin color	1.26 ± 0.09	0.57 ± 0.13^a^	0.63 ± 0.09^a^
Skin elasticity	2.10 ± 0.13	0.38 ± 0.12^a^	0.54 ± 0.17^a^
Extent of disease	2.15 ± 0.44	0.57 ± 0.08^a^	0.72 ± 0.14^a^
Cattanco total points	8.02 ± 1.22	2.21 ± 0.70^a^	2.61 ± 0.5^a^

Compared with pre-operation.

^a^ *P *< 0.05.

### 3.3. Complications and recurrence after surgery

No local burns or blisters were observed after surgery, but one case of a superficial ulcer, one case of mild vulvar swelling, and one case of minor bleeding from the vulva were observed, with an overall complication incidence of 15% (3/20). Superficial ulcers were disinfected with povidone-iodine and gradually healed; vulvar swelling disappeared after applying 10% sodium chloride gauze; vulvar hemorrhage did not recur after applying local pressure with gauze. The complications were relieved after the above prompt intervention. No recurrence was observed among the 20 children 3 months after surgery. However, one case of recurrence was detected 6 months after the procedure. This was successfully treated with external episival application of interferon, resulting in a recurrence rate of 5% (1/20).

## 4. Discussion

The exact mechanism of VLS development is not fully understood, but most scholars believe that its formation is implicated in immune system abnormalities, estrogenic changes, and genetic factors of patients (13). Research has indicated that in patients with VLS, the cellular immune responses, including T-lymphocyte-mediated autoimmune responses, may be abnormal, giving rise to lesions induced by damage to tissues and inflammatory responses (14). VLS may also be induced by immune abnormalities due to autoimmune diseases, such as autoimmune thyroid diseases and rheumatoid arthritis. In cases of decreased estrogen levels, the fragility and vulnerability of the vulvar skin may increase, which in turn facilitates the formation of lesions. One study reported a possible genetic component of VLS, and family history and genetic factors may increase the risk of disease development (15). In this study, two children had immune diseases, and five children had a family history of VLS. In addition, Li Q (16) pointed out that first-degree relatives of patients with VLS have a 12% probability of developing VLS, and the probability of having VLS and skin diseases is 18.9%; however, this was not confirmed in the present study because of the limited sample size.

Currently, the main clinical treatments for VLS are general treatment, drug therapy, physical therapy, and operative therapy. General treatments like cod liver oil ointment and vitamin E cream serve as long-term maintenance therapies. They improve the local skin barrier function and provide short-term relief from symptoms such as vulva dryness. Additionally, medications such as topical steroid creams or emulsions (e.g., adrenocorticotropic hormones) can reduce the symptoms of inflammation, redness, swelling, and itching, but the efficacy is inconsistent, and these treatments are associated with a risk of drug allergies, hormone dependence, and recurrence (17). Washing with warm water and lotion and other physical methods can relieve local itching and discomfort and help maintain local hygiene, but these methods do not fundamentally improve symptoms (18). Surgery can directly remove the diseased cells and tissues to completely cure the disease, and long-term disease control can reduce the risk of recurrence (19).

In this study, children with VLS were treated with clitoris exposure + episioplasty + dermabrasion + PRP injection + chemexfoliation. The results revealed that the Cattanco score of the 20 children was 2.21 ± 0.70) points and 2.61 ± 0.59 points at 3 and 6 months after surgery, respectively, significantly lower than that before surgery (8.02 ± 1.22 points), indicating that the clinical symptoms of VLS were significantly ameliorated, and the therapeutic effect lasted long after surgery. This may be attributed to several factors. First, the surgical approach used in this study improves the blood circulation in the lesion area of VLS by exposing the clitoris and promoting the nutrient supply and metabolism of local tissues, which is conducive to wound healing and lesion relief. Second, the shape and appearance of the vulva are improved by removing VLS tissues through episioplasty. Furthermore, friction and itchiness are reduced after episioplasty, enhancing the comfort of the children. Third, the lesion area is removed by scraping off the VLS tissues via dermabrasion, thereby improving the signs and symptoms of children and reducing itching, pain, and discomfort (20). Moreover, PRP injected into the VLS lesion area releases abundant growth factors and cytokines to promote wound healing and repair as well as tissue regeneration (21). Finally, chemexfoliation (i.e., epidermal exfoliation of the VLS lesion area using a preparation solution containing 88% phenol, croton oil, hexachlorophenol liquid soap, and distilled water) removes the diseased tissues, reduces the inflammatory response, and promotes the growth of new healthy tissues. However, this multimodal treatment also has certain limitations. First, children will be in pain within five days after surgery. Analgesic drugs are needed for controlling the pain of some children and the pain usually disappears after five days. Secondly, the skin color of the vulva after surgery is different from normal skin. Finally, through short-term clinical follow-up, the effect of this multimodal treatment is positive. However, a long-term follow-up is needed to know whether the long-term effect is also satisfactory.

In summary, the mechanism of VLS development is complex and may involve various factors, such as immune abnormalities and changes in estrogen levels. The surgical treatment regimen applied in this study combines multiple surgical and non-surgical approaches to target different aspects of pathological changes, which was expected to achieve a more comprehensive and effective therapeutic effect. Moreover, the procedures and implementation degree of this surgical treatment regimen can be adjusted to accommodate the specific situation of children, allowing personalized therapeutic strategies. Only three cases of mild complications were observed after surgery, and only one case of recurrence was observed in the six months after surgery. This suggests that the surgical treatment regimen is likely safe and its therapeutic effects may be sustained. It should be noted that the results of this study may have some shortcomings due to condition limitations (i.e., the limited sample size and short follow-up period). Future studies will aim to address these limitations by increasing the sample size and extending the follow-up duration. This will be a part of an enhanced study protocol designed to bolster the scientific validity of the results.

In conclusion, clitoris exposure + episioplasty + dermabrasion + PRP injection + chemexfoliation may represent a safe treatment strategy to effectively improve the clinical symptoms and enhance the quality of life of children with VLS.

## Data Availability

The original contributions presented in the study are included in the article/**Supplementary Material**, further inquiries can be directed to the corresponding author.
